# The integrated theory of carcinogenesis: cancer as dysregulated persistence under chronic systemic stress

**DOI:** 10.3389/fonc.2026.1811760

**Published:** 2026-05-15

**Authors:** Wolfgang Kopp

**Affiliations:** Independent Researcher, Graz, Austria

**Keywords:** cancer metabolism, carcinogenesis, mitochondrial regulation, oxidative stress, stem-like persistence, systemic stress, UCP2 (uncoupling protein 2)

## Abstract

Cancer may arise less from broken genes than from a collapse of regulatory control. Mutation-based models do not fully explain why normal tissues tolerate large mutational burdens, why some tumors lack recurrent drivers, or why malignant nuclei can be reprogrammed in healthy cytoplasm. This article proposes the Integrated Theory of Carcinogenesis (ITC), which reframes cancer as dysregulated persistence under chronic systemic stress. A central mediator in this model is mitochondrial uncoupling protein 2 (UCP2), a stress-responsive regulator of metabolism and redox balance. Sustained oxidative, inflammatory, metabolic, and neurohormonal stress is proposed to preserve UCP2 expression, suppress differentiation, resist apoptosis, and stabilize stem-like states. Mutations remain an important part of the narrative; however, within the ITC they are interpreted mainly as downstream consequences or context-dependent accelerators within a destabilized regulatory landscape. By positioning UCP2 as a central molecular hinge between systemic dysregulation and malignant transformation, the ITC offers a unifying explanation for longstanding anomalies and outlines testable paths toward prevention and therapy, including metabolic and lifestyle interventions.

## Introduction

What causes cancer? This question has framed more than a century of biomedical debate. For most of the 20th century, the somatic mutation theory (SMT) offered a simple answer: cancer is the inevitable consequence of mutations accumulating in oncogenes, tumor suppressors, and DNA repair genes (1). So deeply did this view take root that it often became shorthand for “the truth” of cancer biology.

And yet, the story has never been quite so tidy. Normal tissues can be riddled with mutations yet remain benign (2, 3). Some aggressive tumors appear with few, if any, consistent drivers (4, 5). In classic cytoplasmic transfer experiments, malignant behavior could be suppressed by healthy cytoplasm — and conversely, normal nuclei could adopt malignant traits when placed into dysregulated cytoplasm (6, 44). Even precision therapies that neatly target mutations often fall short of durable cures (7). Viral oncogenesis complicates the picture further: viruses such as HPV, HBV, and EBV rarely cause cancer by mutating DNA directly (8).

If mutations cannot fully explain cancer’s origin, what can? An alternative view—one with a surprisingly long intellectual lineage—casts cancer not primarily as a genetic accident but as a failure of regulation. As early as the 19th century, Virchow characterized cancer as “abnormal tissue growth, “ not a fundamentally new cell type. Modern conceptual frameworks echo this regulatory perspective. Tissue Organization Field Theory, for instance, emphasizes disrupted architecture and altered cell–cell communication (9), while metabolic models rooted in Warburg’s observations highlight mitochondrial dysfunction and impaired energy signaling (10, 11).

Here, we extend these ideas into the Integrated Theory of Carcinogenesis (ITC). The ITC proposes that malignant transformation arises when stem and progenitor cells—normally transient and tightly controlled—become pathologically stabilized. Lifestyle-associated exposures disrupt core physiological systems, generating chronic systemic stress: a persistent imbalance across oxidative, inflammatory, metabolic, and neurohormonal pathways. Over time, this stressed milieu reshapes stem cell niches and lowers the threshold for malignant transformation. At the center of this process lies mitochondrial uncoupling protein 2 (UCP2). Chronic systemic stress drives sustained UCP2 expression, which disrupts mitochondrial redox signaling, blocks differentiation, and enables stem-like persistence. In this framework, mutations remain part of the narrative but are interpreted mainly as downstream modifiers — context-dependent accelerators within a destabilized regulatory landscape rather than primary initiating events.

**Figure 1:**
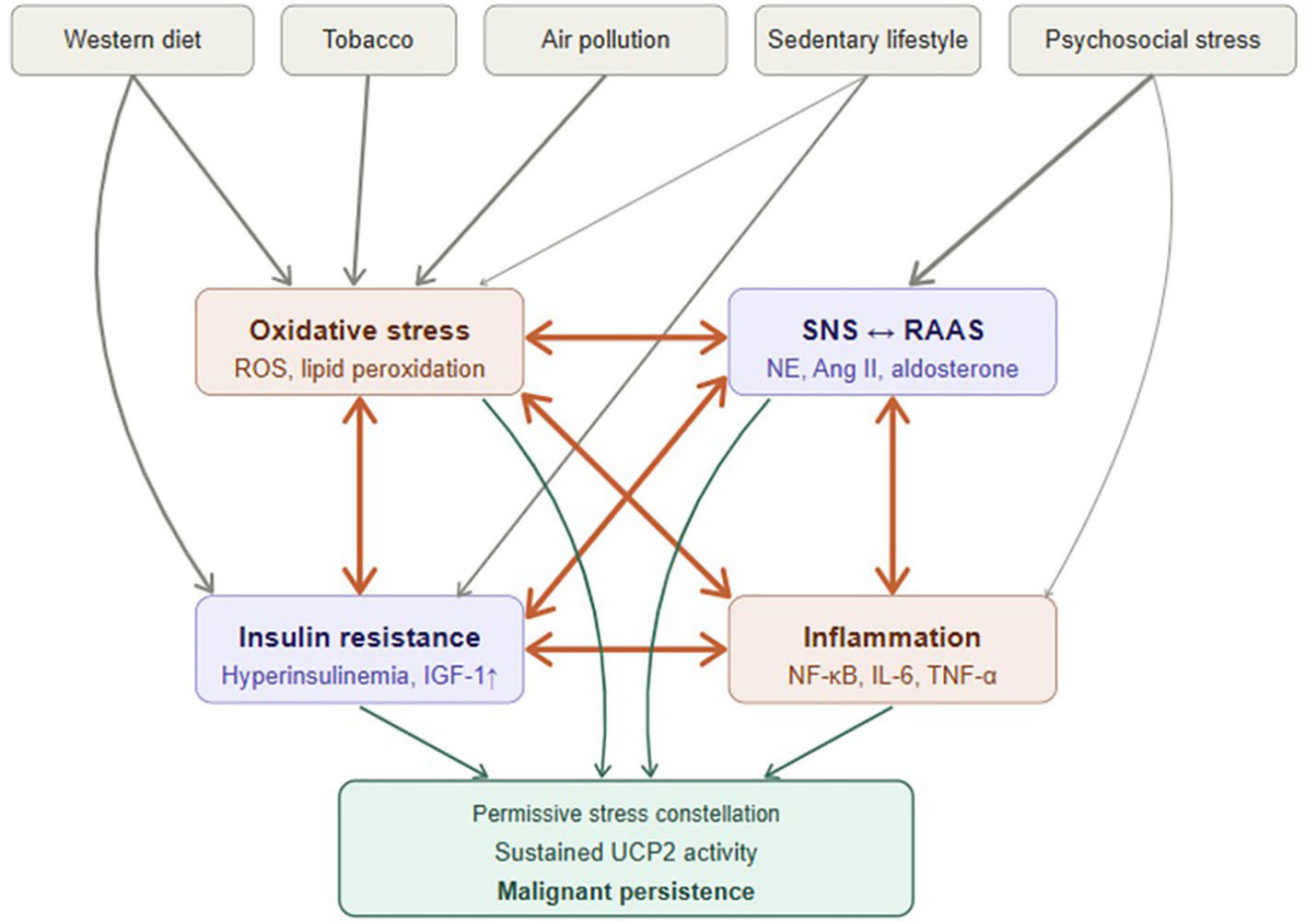
Lifestyle dysregulation as a self-amplifying systemic stress network. Modern lifestyle exposures converge on four interconnected stress domains: oxidative stress, inflammation, insulin resistance/hyperinsulinemia, and neurohormonal activation (SNS/RAAS). These axes mutually amplify each other, forming a self-sustaining network that, under permissive conditions, may drive sustained UCP2 activity and malignant persistence. Sustained UCP2 activity is not proposed to follow automatically from stem cell activation or systemic stress exposure. Rather, it is predicted to emerge only under a permissive constellation of chronic, unresolved stress signals, impaired regulatory counterbalance, and local niche vulnerability — a comparatively rare outcome given the high frequency of physiological stem and progenitor cell activation throughout life. Arrow weight reflects relationship strength based on published evidence. Gray boxes = lifestyle exposures; amber = oxidative/inflammatory nodes; purple = metabolic/neurohormonal nodes. Bidirectional arrows indicate mutual amplification. Conceptually derived from Kopp (2022) (13), licensed under CC BY; substantially revised and extended.

**Figure 2:**
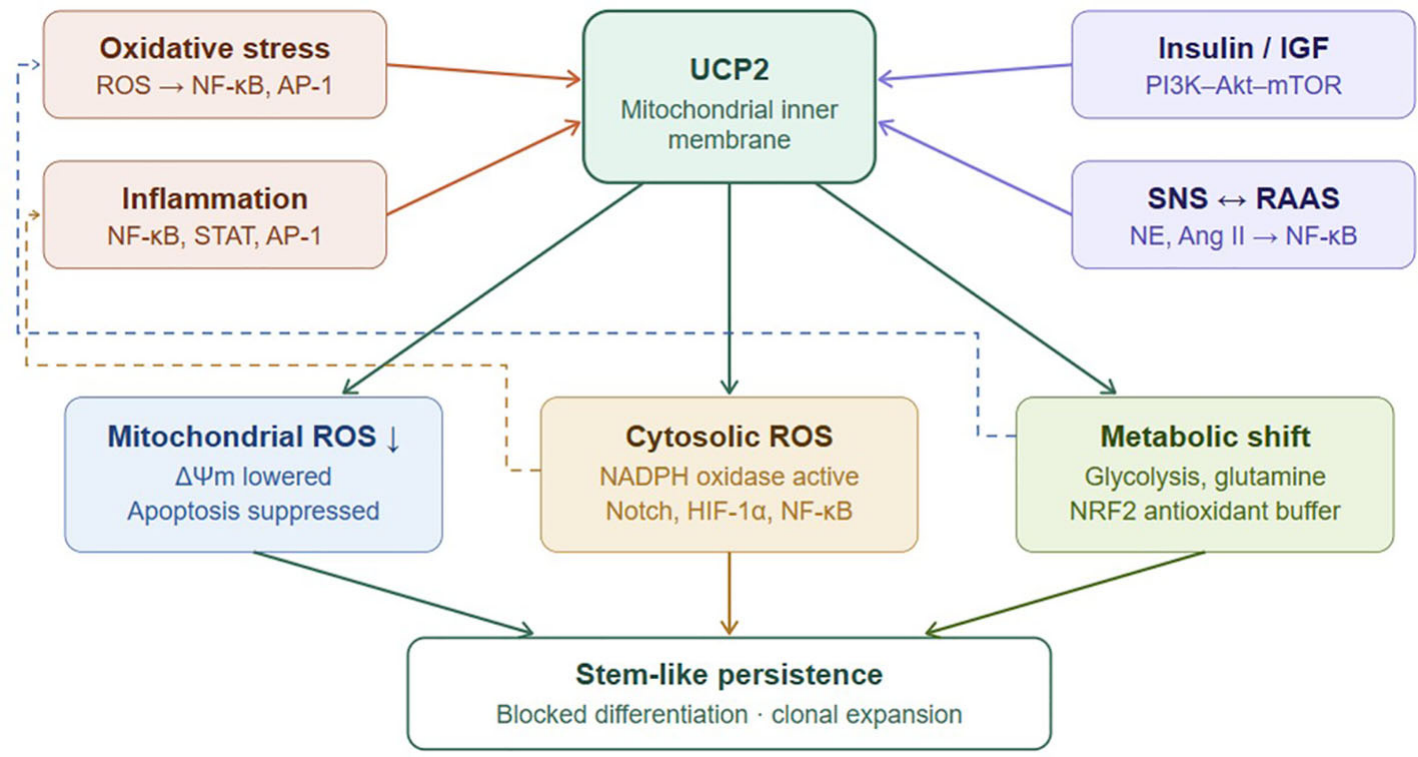
UCP2 as a redox-metabolic hub integrating systemic stress inputs. Oxidative/inflammatory stress signals (coral) and metabolic/neurohormonal inputs (purple) converge on UCP2 (teal) at the mitochondrial inner membrane. Sustained UCP2 activity produces three compartment-specific outputs: suppression of mitochondrial ROS with lowered ΔΨm and blocked apoptosis (blue); preservation of cytosolic ROS pulses activating Notch, HIF-1α, and NF-κB (amber); and metabolic reprogramming toward glycolysis, glutamine metabolism, and NRF2-dependent antioxidant buffering (green). Together, these outputs stabilize stem-like persistence. Solid arrows = direct effects; dashed arrows = feedback loops. Key: decoupled redox compartmentalization — mtROS suppressed while cytosolic ROS pulses persist — underlies the pro-survival signaling that maintains the malignant state.

**Figure 3:**
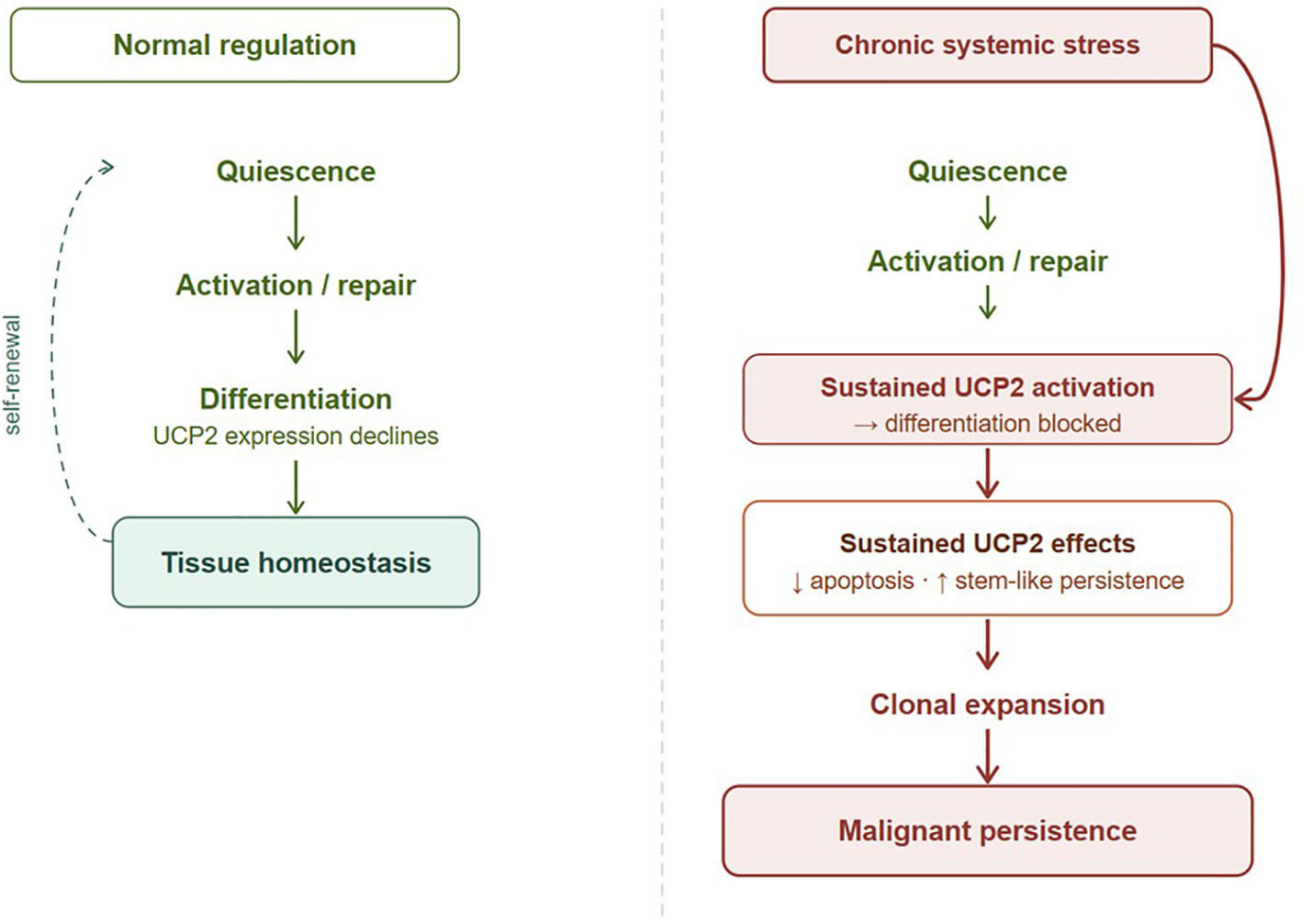
UCP2-mediated stem cell fate under normal versus chronic stress conditions. Left: under normal regulation, stem and progenitor cells progress through quiescence, activation/repair, and differentiation — accompanied by declining UCP2 expression — resulting in tissue homeostasis. Right: chronic systemic stress sustains UCP2 activity, blocking the differentiation step. This suppresses apoptosis and prevents differentiation commitment, leading to clonal expansion and malignant persistence. The key distinction from normal regulation is the failure of UCP2 to decline at the differentiation checkpoint, converting a transient stem-like state into a self-sustaining malignant program. Chronic systemic stress (indicated by external arrow) acts at the differentiation checkpoint, sustaining UCP2 activity and preventing the normal decline in UCP2 expression that permits differentiation.

In this Hypothesis article, we outline how lifestyle-associated systemic stress forms a reinforcing network (**Figure 1**), how UCP2 may act as a molecular hinge integrating these inputs (**Figure 2**), and how its sustained activity could block differentiation and drive malignant persistence (**Figure 3**). Rather than providing a comprehensive review of individual pathways, this article focuses on integrating established mechanisms into a systems-level framework with testable predictions.

## Lifestyle dysregulation as the driving force of carcinogenesis

Why does cancer surge in societies that adopt modern, industrialized lifestyles? The global increase in cancer incidence among societies that have adopted Western lifestyles strongly suggests that cancer is not merely a genetically predetermined disease but rather a disorder influenced by environmental and behavioral factors (12).

Diet, tobacco, pollution, and chronic psychosocial stress create a systemic state marked by oxidative stress, subclinical inflammation, insulin resistance with compensatory hyperinsulinemia, and overactivation of both the sympathetic nervous system (SNS) and the renin–angiotensin–aldosterone system (RAAS). These pathways are tightly interconnected. These overlapping stresses do not act in isolation, but are interlinked such that activation of one amplifies the others, creating a self-sustaining network of oxidative, inflammatory, metabolic, and neurohormonal imbalances (13, 14, 54) (**Figure 1**). This pattern was previously emphasized in the context of smoking-related disease (13) and later extended to aging and chronic disorders (14). Earlier work also suggested that lifestyle-associated systemic stress could promote carcinogenesis and identified UCP2 as a potential mediator (15). The present framework advances this line of thought by positioning UCP2 as the central hinge within a testable systems-level model—the ITC.

Systematic analyses of more than a dozen of the most common chronic non-communicable diseases—including cardiovascular disease, diabetes, COPD, neurodegenerative disorders, and cancer—have shown a network of oxidative, inflammatory, metabolic, and neurohormonal stress that plays a central role in their pathogenesis (13, 14). But in the context of cancer biology, its importance lies in how these systemic signals converge on stem and progenitor cell niches. Oxidative stress activates inflammatory transcription factors such as nuclear factor kappa-light-chain-enhancer of activated B cells (NF-κB) and activator protein 1 (AP-1), while inflammation recruits immune cells that generate yet more reactive oxygen species (ROS), creating a self-reinforcing loop (16). Insulin resistance prompts hyperinsulinemia, which doubles as a growth factor signal, activating proliferative cascades such as the PI3K–Akt–mTOR and Ras–Raf–MEK–ERK pathways (17–19). Chronic sympathetic activation enhances tumor progression through β-adrenergic signaling, promoting angiogenesis, inflammatory cytokine release, metabolic reprogramming, and immune suppression (20, 21). These effects interact with RAAS activation, which amplifies oxidative and inflammatory stress and further reshapes the tumor microenvironment (22).

Taken together, these interconnected stress systems do not simply add risk; they reshape the biological landscape. Instead of maintaining equilibrium, they establish a chronic pro-proliferative, pro-inflammatory environment—a fertile ground for malignant transformation. These exposures are not isolated insults but form an interconnected network of oxidative, inflammatory, metabolic, and neurohormonal stressors (**Figure 1**).

If cancer is rooted in dysregulation, what molecule could bridge systemic stress and stem-like persistence? We propose mitochondrial UCP2. The systemic role of UCP2 in stress-related disease is already well established. Beyond its implication in cancer, UCP2 overexpression has been documented in diabetes, obesity, atherosclerosis, and other metabolic disorders characterized by oxidative and inflammatory stress. In these conditions, UCP2 serves as a key regulator of mitochondrial efficiency and ROS homeostasis, linking metabolic overload to impaired cellular signaling (23). This broader evidence reinforces the view that UCP2 functions as a nodal integrator of systemic stress—a molecule that connects metabolic dysregulation with inflammatory and redox imbalance across diverse non-communicable diseases. Within the ITC, this same integrative function becomes pathologically stabilized in stem and progenitor cells, driving malignant persistence.

## UCP2 at the crossroads of metabolism, ROS, and stemness

In normal stem cells, UCP2 helps maintain flexibility. By diverting tricarboxylic acid intermediates, it limits oxidative phosphorylation and reinforces glycolysis. This bias preserves an undifferentiated state. At the same time, UCP2 lowers mitochondrial ROS (mtROS), shielding cells from oxidative damage while keeping proliferative capacity intact (24–26).

In tumors, UCP2 is rarely mutated but often overexpressed as an adaptive response to stress. NF-κB and related inflammatory pathways regulate its expression, while chronic oxidative pressure selects for its persistence (27, 28). Clinically, UCP2 abundance has been proposed as a biomarker to guide adjunctive metabolic strategies and treatment stratification (29). Beyond oxidative and inflammatory stress, other systemic drivers reinforce UCP2 activity. Chronic immune activation contributes directly to this reinforcement. Persistent NF-κB, AP-1, and STAT signaling—hallmarks of systemic inflammatory stress—increase NADPH-oxidase–derived ROS and upregulate UCP2 transcription in immune and metabolic tissues (30–32). These inflammatory ROS pulses sustain pro-survival transcriptional programs while stabilizing the redox environment in which UCP2 persists. In this way, immune overactivation acts as an amplifier of the same stress-responsive pathways that promote UCP2-driven metabolic flexibility and stem-like persistence.

Hyperinsulinemia and insulin-like growth factor signaling activate PI3K/Akt/mTOR and MAPK cascades, pathways that dovetail with UCP2-mediated metabolic flexibility (17, 18). Similarly, chronic sympathetic and renin–angiotensin–aldosterone activation heighten NF-κB and AP-1 signaling, converging on transcriptional programs that sustain UCP2 expression (21, 22).

These dynamics are consistent with mechanistic evidence from both early and recent studies. Robbins and Zhao (30) demonstrated that UCP2 does not merely buffer mitochondrial ROS but actively reshapes redox signaling—reducing mitochondrial ROS while sustaining cytosolic ROS–dependent activation of NF-κB, MAPK, and HIF pathways. This redirection promotes proliferation and suppresses differentiation, allowing cells to adapt to chronic oxidative and metabolic stress. More recently, Luby and Alves-Guerra (27) confirmed that UCP2 overexpression is a common feature across many tumor types, driving a metabolic shift toward aerobic glycolysis, inhibiting apoptosis, and fostering chemoresistance. Together, these findings support the concept that persistent UCP2 expression represents a maladaptive stabilization of a stress-response program—transforming transient survival advantages into durable malignant persistence.

The paradox lies in UCP2’s handling of ROS. Mild mitochondrial uncoupling suppresses the mtROS bursts that normally trigger DNA damage and apoptosis, yet cytosolic ROS pulses from NADPH oxidases remain intact, activating Notch and HIF signaling (31, 33). A mechanistic interpretation of this redox partitioning further clarifies UCP2’s potential role. Mild uncoupling lowers the inner mitochondrial membrane potential (ΔΨm), thereby reducing mtROS below the threshold required to activate apoptotic signaling. At the same time, metabolic rerouting toward glycolysis and the pentose phosphate pathway preserves cytosolic NADPH availability and permits NADPH oxidase–derived ROS pulses to persist. Concurrent activation of NRF2-dependent antioxidant programs further stabilizes this redox configuration by enhancing glutathione and thioredoxin buffering systems (34). Together, these processes may suppress mitochondria-driven death signals while maintaining cytosolic pro-survival pathways, effectively reshaping redox topology under chronic stress conditions (30, 31, 34).

Antioxidant defenses reinforce this state. NRF2-dependent programs upregulate glutathione and thioredoxin buffering systems, while the pentose phosphate pathway maintains high NADPH/NADP^+^ ratios—together providing resilience against oxidative pressure (34, 56). At the same time, nutritional and metabolic imbalances exacerbate oxidative stress, establishing a link between diet, redox control, and cancer initiation (35). Through these combined mechanisms, UCP2 not only suppresses lethal oxidative injury but integrates systemic metabolic cues into a persistent, stem-like program. Mitochondrial network remodeling under stress further supports this systems-level view. Recent work has shown that cancer cells exposed to acidic microenvironments—conditions characteristic of metabolically stressed tumors—respond by fusing their mitochondria into extensive networks, thereby increasing energy output and survival capacity (36). This structural reorganization mirrors the functional adaptation mediated by UCP2: both serve to buffer oxidative and metabolic stress by optimizing energy efficiency and modulating ROS signaling. Within the framework of the ITC, such mitochondrial plasticity exemplifies how chronic stress induces stable, maladaptive states of survival. What begins as a transient adaptation to environmental pressure becomes a self-perpetuating architecture of persistence—linking systemic dysregulation to the cellular machinery of malignancy.

Through this integration of stress inputs and metabolic outputs, UCP2 is proposed to occupy a central position linking systemic stress to stem-like persistence (**Figure 2**).

## From stem cells to cancer: mechanisms of malignant transformation

Healthy tissues depend on stem and progenitor cells walking a narrow path. Quiescence preserves stability, activation enables repair, and differentiation provides the counterweight that keeps growth in check (37, 38).

Stem cell activation is, in itself, a normal and necessary event. Tissue injury, local inflammation, and routine cell turnover all trigger stem and progenitor cells to leave quiescence and enter the cell cycle. Under healthy conditions, this activation is transient: the cell divides, differentiates, and restores tissue homeostasis. The process is self-limiting because the signals that drive activation also, in time, permit the differentiation switch to engage. What the ITC proposes is not that chronic systemic stress initiates stem cell activation—this happens continuously and physiologically throughout life—but that a chronically dysregulated milieu intercepts the activated state and prevents its resolution. The cell that was activated for a routine purpose cannot complete the journey to differentiation. It remains in a proliferative, undifferentiated state—not because of a genetic accident, but because the environment in which it finds itself no longer permits the return to order.

Given the high frequency of physiological stem and progenitor cell activation throughout life, malignant transformation is unlikely to result from activation alone. The ITC therefore predicts that cancer emerges only when chronic systemic stress coincides with local niche vulnerability and failure of regulatory resolution, creating a permissive constellation in which UCP2-dependent stem-like persistence becomes stabilized. This framing explains why cancer, despite the ubiquity of stem cell activation and stress exposure, remains a relatively rare outcome: it requires not a single trigger, but the simultaneous failure of multiple independent regulatory layers.

These considerations generate additional testable predictions, complementing those outlined in **Box 1**: UCP2-dependent stem-like persistence should require the co-occurrence of multiple chronic stress inputs rather than any single stressor alone, and should be reversible upon restoration of regulatory balance. These predictions are addressable using organoid models, longitudinal tissue profiling, and stepwise stress-induction paradigms.

Box 1 **testable predictions of the integrated theory of carcinogenesis**

• UCP2 upregulation precedes malignant transformation.

In tissues exposed to chronic systemic stress (e.g., obesity, chronic inflammation), stem/progenitor compartments should exhibit elevated UCP2 expression prior to histologic dysplasia or invasion. This prediction can be tested using longitudinal tissue sampling combined with single-cell or spatial transcriptomic/proteomic profiling of UCP2 alongside differentiation markers.

• Systemic stress indices correlate with UCP2 expression.

Composite measures integrating inflammatory markers (e.g., IL-6, CRP), oxidative stress parameters, insulin/IGF activity, and SNS/RAAS activation should correlate with tissue UCP2 levels in a dose-dependent manner across disease stages.

• UCP2 inhibition restores mitochondrial ROS signaling and differentiation.

Genetic or pharmacologic suppression of UCP2 in stress-conditioned premalignant or tumor stem-like cells is predicted to increase mitochondrial ROS, shift metabolic flux toward oxidative phosphorylation, and reactivate differentiation programs and/or apoptotic signaling. Readouts should include compartment-specific ROS measurements, OXPHOS/glycolysis balance, and lineage-specific differentiation markers.

• Redox compartmentalization is required for malignant persistence.

The malignant phenotype should depend on suppressed mtROS combined with preserved cytosolic ROS signaling. Experimental perturbations that restore mitochondrial ROS thresholds or disrupt cytosolic ROS–dependent NF-κB/HIF activity are predicted to destabilize stem-like persistence.

• Stemness programs depend on sustained UCP2 activity.

Under chronic stress conditions, maintenance of stemness signatures (e.g., OCT4, SOX2, NANOG or tissue-specific equivalents) should require active UCP2. UCP2 loss is predicted to reduce self-renewal capacity and promote differentiation commitment in organoid or lineage-tracing models.

Stringent functional test: In nucleus–cytoplasm transfer systems, UCP2 silencing in malignant cytoplasm is predicted to reduce phenotypic capture and destabilize malignant persistence.

These predictions collectively define experimentally tractable routes to evaluate the ITC and distinguish it from mutation-centric models of carcinogenesis.

Chronic immune activation further destabilizes this balance. Cytokine-driven activation of NF-κB, STAT3, and AP-1 reshapes stem-cell niches by amplifying ROS flux, sustaining proliferative and anti-apoptotic signaling, and inducing stromal and vascular remodeling. These pathways inhibit differentiation and promote the survival of undifferentiated cells, aligning with well-established models of inflammation-associated carcinogenesis (39). In this setting, immune overactivation cooperates with UCP2-mediated redox partitioning to stabilize stem-like persistence under systemic stress.

UCP2 normally declines as cells commit to differentiation, allowing the shift toward oxidative metabolism (24). Under chronic systemic stress, this downregulation fails. Inflammation, microenvironmental remodeling, and vascular changes generate persistent ROS- and NRF2-dependent stress signaling that favors sustained UCP2 expression and blocks the differentiation switch (31, 34).

The outcome is “blocked differentiation”: stem-like cells resist apoptosis and expand clonally, supported by metabolic and antioxidant adaptations that make the state durable rather than transient. Beyond oxidative and inflammatory cues, other systemic drivers reinforce this persistence. Hyperinsulinemia and IGF signaling provide proliferative drive through PI3K–Akt–mTOR and Ras–MAPK cascades (17–19). Beyond proliferative drive, insulin and IGF signaling reinforce stem-like traits. They promote self-renewal, EMT, and therapy resistance by intersecting with Wnt/β-catenin and other CSC pathways (40, 55). Chronic sympathetic activity and RAAS signaling further amplify angiogenesis, cytokine release, and vascular remodeling—factors that protect undifferentiated cells and favor malignant progression (20–22, 39, 41).

At the tissue level, these overlapping stresses magnify the effect. Chronic inflammation, insulin resistance, and vascular remodeling not only sustain proliferation but also foster angiogenesis, blunt immune surveillance, and tilt the microenvironment toward malignant persistence (17, 39, 42).

Taken together, these insights support an ITC that links lifestyle exposures, chronic systemic stress, and UCP2-mediated cellular persistence (**Figure 3**). This persistence echoes embryonic programs: many hallmarks of cancer parallel embryo-fetal development, including sustained proliferation, resistance to apoptosis, angiogenesis, and invasion (43, 57). Embryonic stem cells and tumors also share certain gene expression patterns and fetal markers such as alpha-fetoprotein. While these similarities do not imply identity, they suggest that cancer may represent the pathological persistence of developmental traits outside their normal temporal and tissue context (43).

## Discussion: UCP2, stem cells, and a systems-level theory of cancer

The ITC is not intended to replace mutation-based models but to complement them by defining the systemic conditions under which they become biologically decisive. It does not reject genetic, tissue organization, or metabolic theories of cancer but seeks to reframe their relative roles within a systems-level perspective. The ITC does not deny the sufficiency of specific driver mutations in certain contexts but proposes that systemic stress defines the permissive landscape in which such drivers become transformative. While the SMT has provided a powerful genetic framework for understanding tumorigenesis, the ITC complements this view by positioning systemic stress and regulatory failure as upstream contextual determinants, with mutations acting mainly as context-dependent modifiers. Tissue Organization Field Theory focuses on disrupted architecture and cell–cell communication (9); the ITC is consistent with this view but highlights UCP2-mediated metabolic and redox control as a molecular conduit between systemic stress and altered tissue organization. Metabolic theories rooted in Warburg’s observations underscore mitochondrial dysfunction (10, 11); the ITC incorporates this perspective, proposing that UCP2-driven redox compartmentalization and metabolic flexibility stabilize stem-like persistence under chronic stress. In this sense, the ITC seeks to integrate, rather than replace, these existing frameworks.

Modern exposures associated with elevated cancer risk can be broadly distinguished by their primary mode of action. Agents such as tobacco smoke and air pollutants carry direct genotoxic potential; however, their systemic effects — including sustained oxidative stress, subclinical inflammation, and neurohormonal dysregulation — likely contribute independently and additively to carcinogenesis beyond mutagenesis alone. Chronic psychosocial stress acts primarily through neurohormonal activation — driving sustained SNS and RAAS activity, which secondarily amplifies oxidative stress and inflammation. Western dietary patterns, by contrast, act predominantly through metabolic dysregulation, promoting insulin resistance, hyperinsulinemia, and oxidative stress. Neither pathway involves meaningful direct genotoxicity, yet both converge on the same downstream stress network, rendering their carcinogenic potential at least partly independent of mutagenic mechanisms. Mounting evidence shows that oxidative stress, low-grade inflammation, insulin/IGF signaling, and neurohormonal activation through the SNS and RAAS are not independent pathways but form an interconnected stress network (13, 14, 16–22, 54). These pathways are well-established; the novelty of the ITC lies not in their individual description but in their integration into a unified systems-level model that positions chronic systemic stress as the upstream permissive context for malignant transformation. In the ITC, this network provides the systemic milieu in which malignant persistence becomes likely (**Figure 1**).

At the cellular level, chronic stress sustains UCP2 expression, reorganizing mitochondrial redox signaling, reinforcing glycolysis and glutamine metabolism, and bolstering antioxidant defenses (23–27, 30, 31, 33, 34). Together, these adaptations stabilize an undifferentiated, stress-resistant state and lower the threshold for malignant transformation (**Figure 2**).

Within this framework, several longstanding anomalies cohere into a single pattern. Normal tissues can harbor high mutational burdens yet remain benign (2, 3), while some aggressive tumors lack highly recurrent point-mutation drivers, as seen in high-risk neuroblastoma and medulloblastoma (4, 5). Malignant nuclei can be reprogrammed to normal behavior when placed into a healthy cytoplasmic and tissue context (6, 44), while normal nuclei adopt malignant traits in dysregulated cytoplasm (45). These classic nuclear transfer experiments suggest that cytoplasmic and microenvironmental cues—rather than nuclear mutations alone—govern malignant potential. Similarly, mutation-targeted therapies often achieve only transient responses (7). Their limited durability is consistent with the ITC view that blocking a single genetic lesion does not restore differentiation or correct the underlying redox and metabolic dysregulation.

Even tumor heterogeneity can be reinterpreted: rather than reflecting genetics alone, it may partly arise from differences in UCP2 activity and redox state, with some subclones remaining partially differentiable and others locked in persistence.

Clinical observations align with this model. Poorly differentiated tumors generally behave more aggressively (46), and aberrant UCP2 overexpression correlates with reduced differentiation, enhanced proliferation, and apoptosis resistance (47). These patterns fit concepts of retrodifferentiation, in which cancer represents the pathological persistence or reactivation of developmental programs outside their normal temporal and tissue context (43).

Epidemiological and comorbidity data further support a systemic view. Patients with cancer frequently present with clusters of chronic conditions such as cardiovascular disease, diabetes, obesity, COPD, hypertension, or dementia (48). These comorbidities reflect shared oxidative, inflammatory, metabolic, and neurohormonal stress pathways (13, 14), mirroring modern Western lifestyle patterns (49). Importantly, these same stresses—driven by diet, inactivity, pollution, tobacco, and psychosocial load—strongly associate with cancer incidence and converge mechanistically on UCP2. Sustained UCP2 expression stabilizes stem-like states, lowers the threshold for malignant conversion (**Figure 3**), and provides a mechanistic link between systemic dysregulation and malignant persistence within the ITC framework.

Spontaneous regression of cancer, though rare, provides an additional line of support (50, 51). Reported cases often follow systemic perturbations such as acute infections, fever, metabolic or hormonal shifts, or tissue injury, and some regressing tumors show histologic evidence of differentiation. Within the ITC framework, such events can be viewed as transient disruptions of a stress-stabilized state—either by reducing chronic systemic stress or by overwhelming UCP2-buffered ROS dynamics through acute immune activation or infection-induced oxidative surges. What appears as an anomaly may therefore represent a natural experiment in releasing the block on apoptosis and differentiation.

Viral oncogenesis fits this perspective as well. Oncogenic viruses impose oxidative, inflammatory, and metabolic pressures (8, 52, 53) that promote UCP2 upregulation and entrench stem-like persistence, rather than acting primarily through direct mutagenesis. Together with the nuclear transfer data and epidemiological patterns, these observations support the central ITC proposition: that cancer arises chiefly from the pathological stabilization of stemness under chronic systemic stress, with UCP2 serving as a molecular hinge between systemic dysregulation and cellular fate.

Several other stress-responsive molecules — including HIF-1α, NRF2, and AMPK — also operate at the intersection of redox signaling, metabolism, and stemness, and might therefore be considered equally plausible candidates for a central integrative role. The ITC does not dismiss their contributions; indeed, NRF2-dependent antioxidant programs and HIF-driven transcription are explicitly incorporated into the proposed mechanism. However, UCP2 occupies a distinct position for three reasons. First, it acts at the mitochondrial inner membrane, directly modulating the ΔΨm and thereby gating the mtROS threshold that separates survival from apoptotic signaling — a function that upstream transcription factors cannot replicate. Second, unlike HIF-1α or NRF2, which are primarily transcriptional effectors activated downstream of ROS, UCP2 simultaneously shapes the ROS landscape that activates them, placing it causally upstream in the redox hierarchy. Third, UCP2 expression is selectively elevated in stem and progenitor compartments and declines upon differentiation (24, 26), making it uniquely positioned to regulate the stemness–differentiation switch. Together, these properties suggest that UCP2 functions not merely as one node among many, but as a metabolic gatekeeper whose activity integrates systemic stress — operating through the shared stress-signalling architecture described elsewhere (54) — into a durable stem-like state. An additional conceptual implication of the ITC is that malignant persistence may represent a metastable redox state. In this state, UCP2-mediated suppression of mitochondrial ROS coexists with sustained cytosolic ROS signaling, maintaining proliferation while preventing apoptosis and differentiation. This configuration is not fixed but dynamically maintained, suggesting that tumor progression reflects the stabilization of a narrow redox window rather than a unidirectional trajectory. Within this framework, spontaneous regression or therapeutic response can be interpreted as a displacement of the system beyond this window: either toward excessive oxidative stress triggering apoptosis, or toward restoration of redox balance permitting differentiation.

This permissive constellation is likely modulated by additional layers of regulation, including the epigenetic state of stem and progenitor cells, local niche conditions within the tissue microenvironment, and cellular redox buffering capacity — factors that together determine whether UCP2-dependent persistence becomes stabilized or resolved, and that may account for the substantial inter-individual variability in cancer risk under comparable stress exposures.

Metabolic approaches to cancer therapy, including ketogenic interventions (11) and related strategies, aim to reduce glucose availability, insulin signaling, and systemic metabolic stress. The observation that such interventions can slow tumor progression or exert measurable biological effects supports the view that tumors are accessible to systemic metabolic modulation — a finding consistent with the ITC’s emphasis on the metabolic stress network as a driver of malignant persistence. However, their ability to induce durable regression has remained limited in clinical practice. Within the framework of the ITC, this may reflect the persistence of a stabilized redox-metabolic state that is maintained not by metabolic inputs alone, but by a broader constellation of interacting stress pathways that are not directly addressed by metabolic interventions. These considerations suggest that effective therapeutic strategies may require coordinated modulation of multiple stress axes, rather than targeting metabolic inputs in isolation.

Limitations must be acknowledged. A central question for the ITC is whether sustained UCP2 expression is causally upstream of malignant transformation or whether it represents an adaptive response to an already dysregulated microenvironment. These possibilities are not mutually exclusive. The ITC does not require UCP2 to be the singular initiating event; rather, it proposes that once chronically sustained by systemic stress, UCP2 activity becomes necessary for the maintenance of stem-like persistence — a distinction between initiation and perpetuation that is itself experimentally testable. Consistent with a necessary maintenance role, experimental silencing of UCP2 restores apoptosis and differentiation in multiple tumor models (47), and the testable predictions outlined in **Box 1** are designed precisely to distinguish adaptive upregulation from causal stabilization of the malignant state. Causality between UCP2 upregulation and malignant initiation remains to be established through prospective lineage-tracing and organoid studies, as outlined in **Box 1**.

The ITC is not intended as a purely conceptual model; it generates specific, testable predictions that can be evaluated experimentally. These predictions provide clear opportunities for falsification and define a research agenda for empirical validation. Key hypotheses and experimental implications are summarized in **Box 1**.

One particularly decisive test of the ITC would involve classic nucleus–cytoplasm transfer experiments. Normally, a normal nucleus placed into malignant cytoplasm can adopt malignant characteristics (45). The ITC predicts that silencing UCP2 in cancer cytoplasm prior to nuclear transfer would substantially reduce or prevent phenotypic capture of a normal nucleus. Conversely, UCP2 suppression after transfer is predicted to destabilize the malignant program, promoting differentiation or apoptosis. Such an experiment would provide a stringent functional test of UCP2 dependency within the ITC framework.

A particularly decisive and clinically transformative prediction concerns reversibility: if malignant persistence depends on chronically sustained UCP2 activity rather than irreversible genetic damage, then restoration of systemic regulatory balance — through reduction of chronic stress inputs, normalization of UCP2 activity, and re-engagement of differentiation programs — should be capable of destabilizing the malignant state. This prediction is consistent with rare but documented cases of spontaneous tumor regression (50, 51) and positions lifestyle-based and stress-axis interventions not merely as preventive measures but as potential therapeutic strategies.

## Conclusion: cancer as dysregulation — beyond a mutation-centric paradigm

Cancer may be more comprehensively understood not solely as the accumulation of mutations but as the pathological stabilization of stemness under chronic systemic stress. Among the central mediators of this process is mitochondrial UCP2, whose sustained activity enables stem and progenitor cells to resist differentiation, evade apoptosis, and persist clonally. This framework resolves long-standing anomalies in cancer biology: the presence of abundant mutations in normal tissues (2, 3), the reprogramming of malignant nuclei in healthy cytoplasm (44, 45), the limited durability of mutation-targeted therapies (7), and the fact that oncogenic viruses can trigger malignant programs without relying on direct mutational hits (8, 52, 53).

The implications are practical. Durable prevention and therapy may depend less on eliminating mutations than on restoring regulation—normalizing UCP2 activity, re-establishing differentiation, and reducing systemic stress. Lifestyle interventions therefore become central, not peripheral, to cancer control. Virchow’s 19th-century view of cancer as disordered growth remains strikingly relevant: the ITC reframes it in molecular terms as a failure of regulation—deeply tied to the way we live, and potentially reversible. Spontaneous regression, although rare, reinforces this perspective by demonstrating that malignant persistence can be destabilized when systemic balance is restored. This perspective reframes cancer as a metastable, stress-stabilized redox state whose persistence may, at least in principle, be therapeutically destabilized.
